# T follicular helper cells in patients with acute schistosomiasis

**DOI:** 10.1186/s13071-016-1602-6

**Published:** 2016-06-06

**Authors:** Yumei Zhang, Yanjuan Wang, Yanyan Jiang, Wei Pan, Hua Liu, Jianhai Yin, Yujuan Shen, Jianping Cao

**Affiliations:** National Institute of Parasitic Diseases, Chinese Center for Disease Control and Prevention, Shanghai, 200025 China; Key Laboratory of Parasite and Vector Biology, MOH, Shanghai, 200025 China; National Center for International Research on Tropical Diseases, Shanghai, 200025 China; WHO Collaborating Center for Tropical Diseases, Shanghai, 200025 China; Department of Pathogenic Biology, Binzhou Medical University, Yantai, 264003 Shandong China; Department of Pathogenic Biology and Immunity, Xuzhou Medical University, Xuzhou, 221004 Jiangsu China

**Keywords:** T follicular helper cells, PD-1, IL-21, Acute schistosomiasis

## Abstract

**Background:**

The role of T follicular helper (Tfh) cells in schistosome infection is not fully defined. In a previous study, a higher frequency of circulating PD-1^+^CXCR5^+^CD4^+^ Tfh cells was observed in patients with chronic schistosomiasis relative to healthy controls (HCs) and it correlated positively with the level of soluble egg antigen (SEA) specific antibodies in serum. However, the function of Tfh cells in patients with acute schistosomiasis remains elusive; this was investigated in the present study.

**Methods:**

The frequency of circulating Tfh cells and the expression of inducible T cell co-stimulator (ICOS), programmed cell death 1 (PD-1) and B cell subsets were analyzed in 12 patients with acute schistosomiasis and 10 HCs by flow cytometry. The expression of Bcl6, c-Maf and IL-21 mRNA were detected by quantitative real-time reverse transcriptase PCR (qRT-PCR). The concentration of serum IL-21 and IgG specific to *Schistosoma japonicum* antigen were then determined by enzyme linked immunosorbent assay (ELISA). Correlations between PD-1^+^CXCR5^+^CD4^+^ Tfh cells, memory B cells and IgG specific to *S. japonicum* were analyzed by Spearman’s rank correlation.

**Results:**

The frequency of PD-1^+^CXCR5^+^CD4^+^ Tfh and memory B cells was increased in acute schistosomiasis patients relative to HCs. Moreover, the levels of IL-21 in serum and the expression of IL-21 mRNA were higher in acute schistosomiasis patients. However, there was no significant correlation between PD-1^+^CXCR5^+^CD4^+^ Tfh cells, memory B cells and IgG specific to *S. japonicum* antigen in patients with acute schistosomiasis.

**Conclusions:**

PD-1^+^CXCR5^+^CD4^+^ Tfh cells in peripheral blood are involved in the immune response of patients with acute schistosomiasis. Understanding the immunological mechanism is helpful for the development of vaccination strategies to control schistosomiasis.

## Background

Schistosomiasis is a serious public health problem in many developing countries. More than 200 million people are infected and 700 million people are at risk of becoming infected [1, 2]. There is no effective anti-schistosome vaccine because of insufficient knowledge of immunological responses to schistosomiasis. Humoral immunity is crucial for successful vaccines. Recent studies reveal Tfh cells are a separate subset of CD4^+^ T cells that are mainly responsible for promoting B cells to undergo proliferation, isotype switching and antibody response [3–5]. Recently, a study showed that Tfh cells were recruited to the liver and upregulated hepatic granuloma formation and liver injury in *S. japonicum*-infected mice [6]. Our previous study showed that Tfh cells might play an important role in the production of specific antibodies in chronic schistosomiasis patients [7], but their function in acute schistosomiasis patients is unknown.

Although Tfh cells in the germinal center (GC) zones are easily detected, it is difficult to obtain lymph nodes or spleen from schistosomiasis patients. Circulating Tfh cells were reported to share some features of GC Tfh cells [8], thus analysis of circulating Tfh cells may be an effective alternative approach to studying GC Tfh cells in humans. In this paper, we determined the characteristics of circulating Tfh cells in acute schistosomiasis patients. PD-1^+^CXCR5^+^CD4^+^ Tfh cells were significantly increased and were involved in the immune response. These data contribute to understanding the role of Tfh cells in acute schistosomiasis and will support vaccine development.

## Methods

### Ethical statement

Ethical approval for this study was obtained from the Ethics Committee of the National Institute of Parasitic Diseases, Chinese Center for Disease Control and Prevention (reference no. 2012–12). This study was conducted according to the guidelines provided in the Declaration of Helsinki and all procedures were approved by the Ethics Review Committee of the National Institute of Parasitic Disease, Chinese Center for Disease Control and Prevention. The objectives and procedures of the study were verbally explained to each participant and written informed consent was obtained.

### Patients and healthy controls

The study was carried out on a total of 22 subjects from Dongting Lake Basin, Hunan Province, China. The profiles of participants is shown in Table 1. The participants included 10 healthy adult controls and 12 acute schistosomiasis patients. Patients were initially screened for *Schistosoma* spp. eggs in feces using the Kato-Katz method [9]. Then IgG specific to soluble egg antigen (SEA) and indirect hemagglutination antibody (IHA) in sera were detected. Acute schistosomiasis patients were diagnosed by positive results of fecal egg, IgG specific to SEA and IHA analyses. They also had a fever or fatigue accompanied by tenderness in the liver region and had been in contact with cercariae in water in the last 3 months. Moreover, they had no history of schistosome infection. Healthy controls did not display clinical symptoms of schistosome infection and produced negative laboratory results. All participants were free from HBV and HCV.

**Table 1:** Participant profiles

No. participant	Gender	Age (years)	Occupation	*Schistosoma* eggs in faeces	IHA^c^	SEA IgG^d^
P1^a^	M	24	Peasant	+	+	+
P2	M	21	Peasant	+	+	+
P3	M	45	Fisherman	+	+	+
P4	M	31	Fisherman	+	+	+
P5	M	51	Fisherman	+	+	+
P6	M	48	Worker	+	+	+
P7	M	48	Fisherman	+	+	+
P8	M	24	Peasant	+	+	+
P9	M	44	Fisherman	+	+	+
P10	M	28	Fisherman	+	+	+
P11	M	48	Fisherman	+	+	+
P12	M	49	Fisherman	+	+	+
H1^b^	M	51	Peasant	–	–	–
H2	M	54	Worker	–	–	–
H3	M	42	Doctor	–	–	–
H4	M	21	Worker	–	–	–
H5	M	52	Worker	–	–	–
H6	M	44	Worker	–	–	–
H7	M	34	Worker	–	–	–
H8	M	28	Peasant	–	–	–
H9	M	45	Doctor	–	–	–
H10	M	52	Peasant	–	–	–

*Abbreviations*: ^a^P 1–12, schistosomiasis patients; ^b^H1–10, healthy controls; ^c^Indirect hemagglutination antibody (IHA); ^d^IgG specific to soluble egg antigen (SEA)

### Flow cytometry analysis

Peripheral blood mononuclear cells (PBMCs) were collected and isolated by Ficoll-density gradient centrifugation. PBMCs were immuno-stained for 30 min with the following antibodies, Tfh cells: CD4-PE, ICOS-FITC (eBioscience, San Diego, CA,USA), CXCR5-APC and PD-1 PE-Cy7 (BioLegend, San Diego, CA, USA); circulating B cells: CD4-FITC, CD19-APC and CD27-PE. The stained cells were then assessed using a CyAn ADP cytometer (Beckman Coulter, Lambertville, NJ, USA) and data were analyzed with FlowJo software.

### RNA extraction and real-time PCR

CD4^+^ T cells from PBMC were purified using human CD4 MicroBeads (Miltenyi Biotec, San Diego, CA,USA) in accordance with the instructions of kit. RNA from CD4^+^ T cells was extracted using Trizol reagent and cDNA was produced using Taqman reverse transcription kits (TaKaRa, Shiga, Japan). Real-time PCR was performed using SYBR Supermix (TaKaRa, Shiga, Japan) and a CFX96 analysis system (Bio-Rad, Richmond, CA, USA). Each sample was amplified in triplicate. The primer sequences were: IL-21 sense 5ʹ-CAC AGA CTA ACA TGC CCT TCA T-3ʹ and antisense 5ʹ-GAA TCT TCA CTT CCG TGT GTT CT-3ʹ; Bcl-6 sense 5ʹ-AAG GCC AGT GAA GCA GAG A-3ʹ, and antisense 5ʹ-CCG ATA GGC CAT GAT GTC T-3ʹ; c-Maf sense 5ʹ-CAA GCT AGA AGC GCC CC-3ʹ, and antisense 5ʹ-AGT TCT GAT GCC ATT CTC CTG-3ʹ; β-actin sense 5ʹ-AGC GAG CAT CCC CCA AAG TT-3ʹ, and antisense 5ʹ-GGG CAC GAA GGC TCA TCA TT-3ʹ. The real-time PCR amplification conditions were: denaturation at 95 °C for 5 min followed by 40 cycles of 95 °C for 10 s and 60 °C for 30 s. Fluorescence values were recorded at 60 °C.

### Detection of IL-21 and IgG specific to *S. japonicum* antigen

The levels of serum IL-21 were analyzed using a human IL-21 ELISA Ready-SET-Go kit (eBioscience, San Diego, CA, USA) according to the manufacturer’s protocol. The levels of serum IgG specific to SEA, adult worm antigen and schistosomula antigen were determined by ELISA. SEA, adult worm antigen and schistosomula antigen were prepared as described previously [10]. Briefly, 96-well plates were coated with 5 μg/ml SEA, adult worm antigen or schistosomula antigen in coating buffer overnight at 4 °C. After washing and blocking, serum from schistosomiasis patients was added and HRP-conjugated goat anti-human IgG was used as the secondary antibody. All samples were analyzed in triplicate using the average absorbance at 450 nm to calculate concentrations.

### Statistical analysis

Statistical differences between two groups were determined by independent t-test or Mann-Whitney U test using SPSS19.0 software. Correlations between groups were analyzed using Spearman’s rank correlation (r_s_). A two-sided *P-*value of <0.05 was considered statistically significant.

## Results

### Clinical characteristics of study subjects

Tweleve acute schistosomiasis patients (aged 38 ± 11.7) and 10 healthy controls (aged 42 ± 11.2) were enrolled in the study. There was no statistically significant difference in age between the acute schistosomiasis patients and healthy controls; all patients were male (*t*_(20)_ = -0.789, *P* = 0.439).

### Characteristics of Tfh cells in acute schistosomiasis patients

**Fig. 1:**
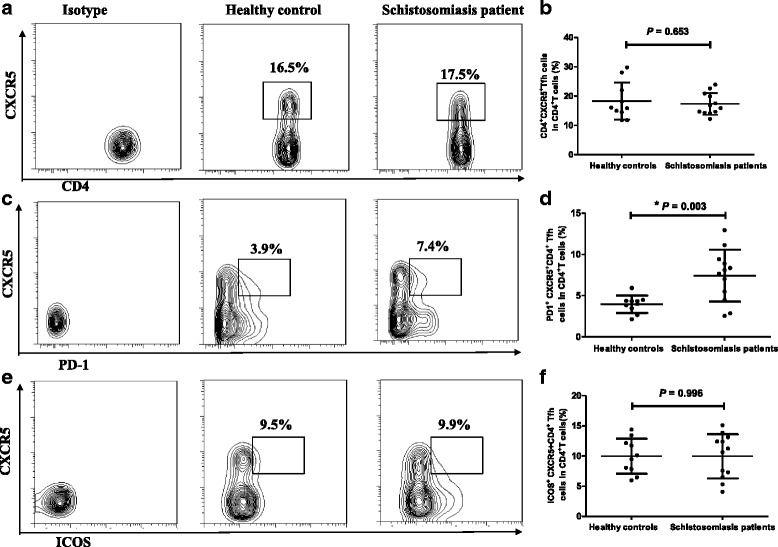
Flow cytometry analysis of Tfh cells in acute schistosomiasis patients and healthy controls. Isotype controls were used to determine positive cells. All of the values were gated on CD4^+^ cells. **a** Values correspond to the percentage of CXCR5^+^CD4^+^ Tfh cells. **b** Comparison of the frequencies of CXCR5^+^CD4^+^ Tfh cells in healthy controls (HC) and schistosomiasis patients. **c** Values correspond to the percentage of PD-1^+^CXCR5^+^CD4^+^ Tfh cells. **d** Comparison of the frequencies of PD-1^+^CXCR5^+^CD4^+^ Tfh cells (*P < 0.01). **e** Values correspond to the percentage of ICOS^+^CXCR5^+^CD4^+^ Tfh cells. **f** Comparison of the frequencies of ICOS^+^CXCR5^+^CD4^+^ Tfh cells

The percentage of circulating CXCR5^+^CD4^+^ Tfh cells among CD4^+^ T cell population was monitored by flow cytometry (Fig. 1a). As shown in Fig. 1b, the frequency of CXCR5^+^CD4^+^ Tfh cells was similar between acute schistosomiasis patients and the HC group. Next we compared activated peripheral Tfh [11, 12] cells (PD-1^+^CXCR5^+^CD4^+^ Tfh and ICOS^+^CXCR5^+^CD4^+^ Tfh cells) (Fig. 1c, e,). There was a significantly higher frequency of PD-1^+^CXCR5^+^CD4^+^ Tfh cells in acute schistosomiasis patients compared with healthy control (Fig. 1d) (*t*_(20)_ = -3.575, *P* = 0.003)*.* However, there was no statistically significant difference in the percentage of ICOS^+^CXCR5^+^CD4^+^ Tfh cells between the acute schistosomiasis and HC groups (Fig. 1f) (*t*_(20)_ = 0.005, *P* = 0.996).

### Increased mRNA expression of IL-21 but not Bcl-6 or c-Maf

**Fig. 2:**
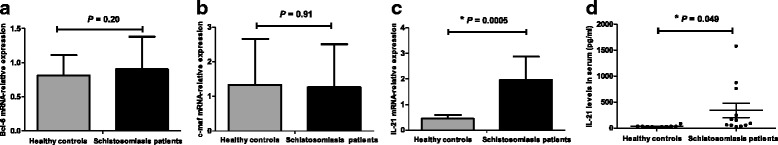
Analysis of transcription factors and IL-21 levels in acute schistosomiasis patients and HCs. Real-time PCR analysis transcription factors and IL-21. **a** Detection of Bcl-6 mRNA expression. **b** Detection of c-Maf mRNA expression. **c** Detection of IL-21 mRNA expression (*P < 0.01). **d** Levels of IL-21 in sera from schistosomiasis patients and HCs (*P < 0.05)

Cytokine IL-21 and transcriptional factors Bcl-6 and c-Maf play critical roles in the differentiation and function of Tfh cells [13–15]. We assessed the expression of Bcl-6, c-Maf and IL-21 mRNA in the CD4^+^ T cells of acute schistosomiasis patients and healthy controls (Fig. 2). Bcl-6 and c-Maf mRNA expression levels in CD4^+^ T cells of patients were similar to those in controls (Fig. 2a, b). IL-21 is a typical cytokine produced by Tfh cells and can enhance production of antibodies by B cells. The mRNA level of IL-21 was higher in patients compared to healthy controls (Fig. 2c) (*t*_(8)_ = -6.791 *P* = 0.0005). Moreover, the level of serum IL-21 was significantly higher in patients than in HCs (Fig. 2d) (*t*_(20)_ = -2.001, *P* = 0.049).

### FACS analysis of different subsets of B cells

**Fig. 3:**
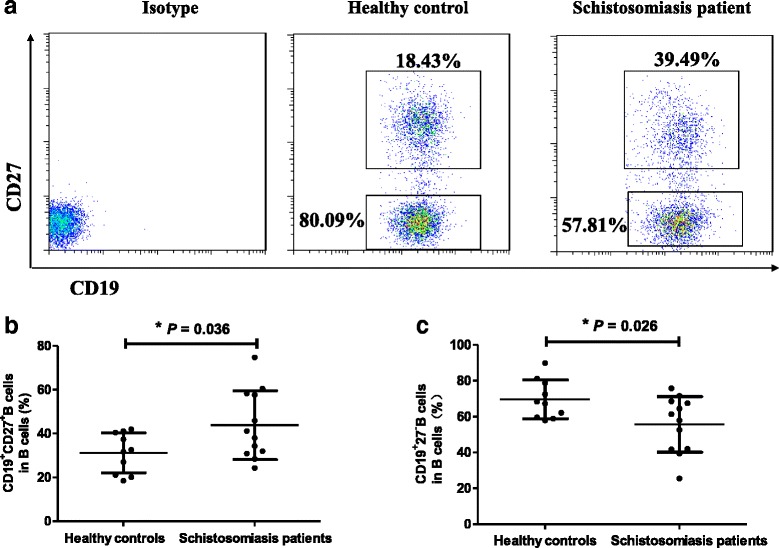
Increased frequencies of peripheral memory B cells in patients with acute schistosomiasis japonica. Isotype controls were used to determine positive cells. All of the values were gated on B cells. **a** Values in the upper and lower right quadrants correspond to the percentage of memory B (CD4^−^CD19^+^CD27^+^) cells and naïve B (CD4^−^CD19^+^CD27^−^) cells among the total B cells, respectively. **b** Correlation between the percentages of memory B cells in HCs and acute schistosomiasis patients (*P < 0.05). **c** The correlation between the percentages of naïve B cells in the two groups (*P < 0.05)

Tfh cells are mainly responsible for assisting B cells to induce antibody response. Therefore, we characterized the frequency of observation of different stages of B cells by flow cytometry (Fig. 3a). As shown in Fig. 3b, the percentage of CD27^+^CD19^+^CD4^−^ memory B cells [16–18] in acute schistosomiasis patients was significantly higher than in HCs (*t*_(20)_ = 2.250, *P* = 0.036). In contrast, the percentage of CD27^−^CD19^+^CD4^−^ naïve B cells [19] in patients was lower than in the HCs (Fig. 3c) (*t*_(20)_ = -2.396, *P* = 0.026).

### Relationship between the percentage of PD-1^+^CXCR5^+^CD4^+^ Tfh cells and other clinical parameters in acute schistosomiasis patients

**Fig. 4:**
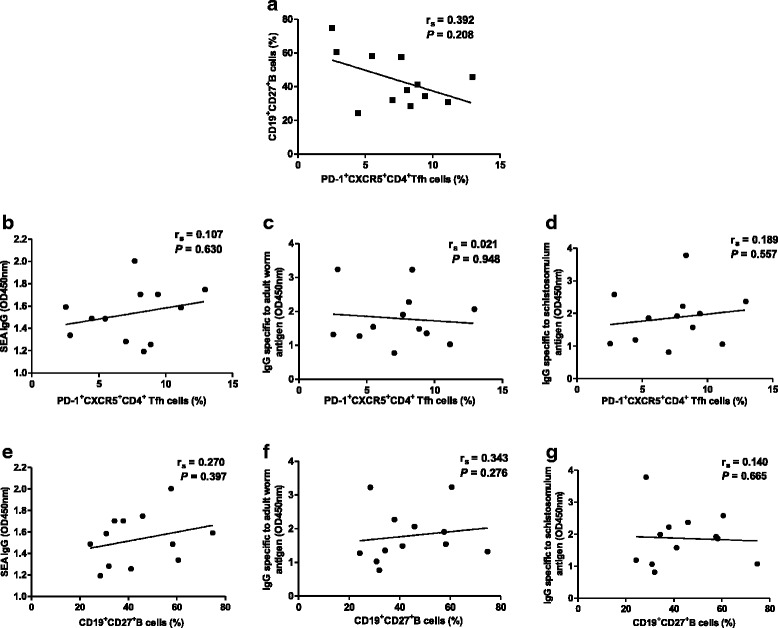
Correlation of circulating Tfh cells, memory B and IgG specific to *Schistosoma japonicum* antigen in schistosomiasis patients. **a** Relationship between memory B cells and the percentage of PD-1^+^CXCR5^+^CD4^+^ Tfh cells. **b** Relationship between the percentage of PD-1^+^CXCR5^+^CD4^+^ Tfh cells and IgG specific to SEA. **c** Relationship between the percentage of PD-1^+^CXCR5^+^CD4^+^ Tfh cells and IgG specific to adult worm antigen. **d** Relationship between the percentage of PD-1^+^CXCR5^+^CD4^+^ Tfh cells and IgG specific to schistosomulum antigen. **e** Relationship between memory B cells and IgG specific to SEA. **f** Relationship between memory B cells and IgG specific to adult worm antigen. **g** Relationship between memory B cells and IgG specific to schistosomula antigen. None of the correlations reached the level of statistical significance

Given Tfh cells can promote B cells to secrete antibodies [20], we determined whether IgG specific to *S. japonicum* antigen correlated with B cells or Tfh cells in patients. There was no significant correlation between the percentages of circulating PD-1^+^CXCR5^+^CD4^+^ Tfh cells and CD27^+^CD19^+^CD4^−^ memory B cells in patients (Fig. 4a) (*r*_s_ = 0.392, *P* = 0.208). There was a tendency towards correlation between the percentage of PD-1^+^CXCR5^+^CD4^+^ Tfh cells and the level of IgG specific to *S. japonicum* antigen (Fig. 4b–d), but statistical significance was not reached (r_s_ = 0.107, *P* = 0.630; *r*_s_ = 0.021, *P* = 0.948; *r*_s_ = 0.189, *P* =0.557). Furthermore, no statistically significant correlation was observed between CD27^+^CD19^+^CD4^−^ memory B cells and the level of IgG specific to *S. japonicum* antigen (Fig. 4e-g) (*r*_s_ = 0.270, *P* = 0.397;* r*_s_ = 0.343, *P* = 0.276; *r*_s_ = 0.140, *P* = 0.665).

## Discussion

At present, praziquantel remains highly effective in treating schistosomiasis. However, drug resistance and decreased susceptibility occur with long-term use of drugs [21]. Therefore, effective vaccines against schistosome infection are necessary. Tfh cells promote antibody production through interaction with B cells [22]. Tfh cells play an important role in long-term humoral immunity and have proven to be one of the important contributors to the effect of successful vaccines [23]. However, the role of Tfh cells in schistosomiasis patients is not well understood and this limits our ability to develop anti-schistosome vaccines. Previous studies revealed an increased frequency of Tfh cells in mice with schistosome infection [6, 24]. Both our previous study and other research focused on the characteristics of peripheral Tfh cells in chronic schistosomiasis patients [7, 25]. However, whether the number of Tfh cells increase in acute schistosomiasis patients remains unknown. The present study thus extended previous work to assess Tfh cells in acute schistosomiasis patients.

No obvious fluctuation in the frequency of CXCR5^+^CD4^+^ Tfh cells was observed between acute schistosomiasis patients and the HC group. To further identify whether this subset of cells plays a role in acute schistosome infection, the expression profiles of two markers associated with Tfh cell function (PD-1, ICOS) were determined. A significantly higher frequency of PD-1^+^CXCR5^+^CD4^+^ Tfh cells was found in acute schistosomiasis patients than in HCs. This might suggest that PD-1^+^ Tfh cells are associated with production of antibodies against the parasite because PD-1 has been demonstrated to regulate the formation of GCs and the survival of B cells [26, 27]. In addition, the relationship between PD-1^+^CXCR5^+^CD4^+^ Tfh cells and IgG specific to *S. japonicum* antigen in patients’ serum was explored, but no statistical correlation was found. In contrast to this result, our previous study of chronic patients showed a positive correlation between PD-1^+^CXCR5^+^CD4^+^ Tfh cells and IgG specific to SEA [7]. This discrepancy might be due to distinguished immune responses in chronic and acute stages of the disease, but the specific mechanism requires further research. Our previous study found increased expression of ICOS in the Tfh cells of chronic schistosomiasis patients relative to HCs [7]. However, there was no significant difference between the levels of these two parameters in acute patients and HCs. In a mouse model, it was found that the expression of ICOS in Tfh cells was involved in the formation of liver granuloma [6]. Compared with the control group, liver granuloma significantly enlarged in the chronic phase, but not in the acute phase in the ICOS transgenic mouse model infected with *S. japonicum* [28]. Thus we speculate that the livers of chronic schistosomiasis patients have varying degrees of fibrosis induced by ICOS expression. The expression of ICOS might not be detectably upregulated in acute schistosomiasis patients because their livers have only slight damage.

The expression of IL-21 mRNA and the IL-21 levels in serum were significantly higher in acute schistosomiasis patients than in HCs. IL-21 is a critical cytokine released by Tfh cells, which can help with Tfh development and antibody production [29, 30]. The upregulated expression of IL-21 might imply an enhanced function of Tfh cells in acute phase infection. Unlike the situation in chronic patients, there was no significant positive correlation between PD-1^+^CXCR5^+^CD4^+^ Tfh cells and levels of serum IL-21. Moreover, the mRNA expression of the transcription factors Bcl-6 and c-Maf, which are essential for Tfh cell differentiation, also showed no apparent upregulation. The latter is in accordance with our previous study in chronic schistosomiasis patients. It has been demonstrated that GC Tfh cells expressed high levels of Bcl-6 while circulating Tfh cells failed to express increased levels of Bcl-6 [20].

A higher percentage of memory B cells was observed in acute schistosomiasis patients relative to HCs. This is in accordance with the previous observation [25] that memory B cells and plasma cells were increased in schistosomiasis patients. However, there was no statistical correlation between memory B cells and levels of IgG specific to *S. japonicum* antigen. It may be that other subsets of B cells, but not memory B cells, correlate with IgG specific to *S. japonicum* antigen in schistosomiasis patients.

## Conclusions

This study assessed the changes in circulating Tfh cells and B cells in acute schistosomiasis patients. Better understanding the differences in immune mechanisms between acute and chronic schistosomiasis patients will be helpful for vaccine design to prevent schistosomiasis.

## Abbreviations

ELISA, enzyme-linked immunosorbent assay; GC, germinal centers; HC, healthy control; IHA, indirect hemagglutination antibody; PBMCs, peripheral blood mononuclear cells; SEA, soluble egg antigens; Tfh, T follicular helper
